# L-Carnitine Tartrate Downregulates the ACE2 Receptor and Limits SARS-CoV-2 Infection

**DOI:** 10.3390/nu13041297

**Published:** 2021-04-14

**Authors:** Aouatef Bellamine, Tram N. Q. Pham, Jaspreet Jain, Jacob Wilson, Kazim Sahin, Frederic Dallaire, Nabil G. Seidah, Shane Durkee, Katarina Radošević, Éric A. Cohen

**Affiliations:** 1 Lonza Consumer Health, Morristown, NJ 07960, USA; shane.durkee@lonza.com; 2 Institut de Recherche Clinique de Montreal, Montreal, QC H2W1R7, Canada; Tram.Pham@ircm.qc.ca (T.N.Q.P.); Jaspreet.Jain@ircm.qc.ca (J.J.); frederic.dallaire@ircm.qc.ca (F.D.); Nabil.Seidah@ircm.qc.ca (N.G.S.); 3 Applied Science and Performance Institute, Tampa, FL 33607, USA; jwilson@theaspi.com; 4 Department of Animal Nutrition, Faculty of Veterinary Medicine, Firat University, Elazig 23119, Turkey; nsahinkm@yahoo.com; 5 Biologics R&D, Lonza Pharma & Biotech, 4057 Basel, Switzerland; katarina.radosevic@lonza.com; 6 Department of Microbiology, Infectiology and Immunology, Université de Montréal, Montreal, QC H3T 1J4, Canada

**Keywords:** L-carnitine, SARS-CoV-2, COVID-19, exercise, inflammation, ACE-2, Furin, TMPRSS2

## Abstract

Severe acute respiratory syndrome coronavirus 2 (SARS-CoV-2) has been responsible for one of the worst pandemics in modern history. Several prevention and treatment strategies have been designed and evaluated in recent months either through the repurposing of existing treatments or the development of new drugs and vaccines. In this study, we show that L-carnitine tartrate supplementation in humans and rodents led to significant decreases of key host dependency factors, notably angiotensin-converting enzyme 2 (ACE2), transmembrane protease serine 2 (TMPRSS2), and Furin, which are responsible for viral attachment, viral spike S-protein cleavage, and priming for viral fusion and entry. Interestingly, pre-treatment of Calu-3, human lung epithelial cells, with L-carnitine tartrate led to a significant and dose-dependent inhibition of the infection by SARS-CoV-2. Infection inhibition coincided with a significant decrease in ACE2 mRNA expression levels. These data suggest that L-carnitine tartrate should be tested with appropriate trials in humans for the possibility to limit SARS-CoV-2 infection.

## 1. Introduction

Severe acute respiratory syndrome coronavirus (SARS-CoV-1 and SARS-CoV-2) is responsible for the SARS epidemic from 2002 to 2004 and more recently for the coronavirus disease 2019 (COVID-19) pandemic outbreak initially detected in December 2019 in Wuhan China [1]. The rapid spread of the disease has affected more than 120 million people and caused over 2.6 million deaths in 220 countries as per mid-March of 2021 [2]. SARS-CoV-2 is an airborne virus that affects mainly the lungs and the upper respiratory system [2], leading ultimately to lung injury, respiratory distress, and death in severe cases.

According to recent data released, there are 237 known vaccine candidates globally, of which 64 are in clinical evaluation and nine are in their phase III clinical stage [3,4]. A few vaccines starting with Pfizer-BioNTech and Moderna’s mRNA-based vaccines and more recently Johnson & Johnson’s single-shot adenovirus vector-based vaccine have been approved for adult use [5]. As a result of the urgency of the situation, accelerated clinical development paths have been followed [3,6] and resulted in decreased vaccine acceptance [6]. In addition to the vaccines, there are a number of other preventive and therapeutic strategies being developed, including antibodies [7,8] and antiviral drugs [9,10].

The SARS-CoV-2 spike protein, which is the main target for the development of vaccines and other therapeutics, mediates viral attachment to cells through binding to the angiotensin-converting enzyme 2 (ACE2) receptor [11,12]. Upon cleavage of a spike by cellular proteases including Furin and transmembrane protease serine 2 (TMPRSS2), the virus enters via a fusion process and replicates in certain target cells including lung epithelial cells [11,12]. In human airway cells, spike cleavage at the S1/S2 position by Furin and at the S2’ position by Furin and TMPRSS2 prime the viral protein and allow viral fusion and entry to occur [13,14]. These proteases are currently being investigated as potential targets for a number of SARS-CoV-2 drug therapies [14].

The physiological role of ACE2 is to lower blood pressure and counteract inflammation by converting pro-inflammatory Angiotensin II to anti-inflammatory Angiotensin (1–7) [14]. The conversion from Angiotensin I to Angiotensin II is mediated by ACE1. ACE inhibitors are commonly prescribed and widely used in clinical practice as standard therapy, in mono or polytherapy, for the management of hypertension and heart failure [15]. Increased ACE1 or decreased ACE2 may reflect an increased inflammatory state [16]. Therefore, shifting the balance toward a lower and hence a more anti-inflammatory ACE1/ACE2 ratio is essential when considering therapies targeting ACE2, particularly since a peaking in the inflammatory response and subsequent cytokine storm have been reported in advanced diseased COVID-19 patients [17].

L-carnitine is an amino-acid-like molecule used mainly as a nutritional supplement for a variety of health benefits [18,19,20]. L-carnitine’s basic role is to transport fatty acids to the mitochondrial matrix, making them available for beta-oxidation and energy generation through the Krebs cycle [21]. It is also a powerful anti-oxidant and anti-inflammatory mediator [22,23]. At the tissue level, L-carnitine accumulates mainly in the muscle, heart, and lung tissues [24]. The elderly and people with chronic diseases (e.g., obesity, diabetes, hypertension, and cardiovascular diseases) exhibit significantly lower levels of L-carnitine in their tissues compared to the healthy population [25]. These compromised populations are prone to having chronic inflammation, which is a condition that is shown to be mitigated by L-carnitine supplementation [23]. In addition, L-carnitine has been shown to reduce ACE1 levels in a hypertensive rodent model [26], and in the context of viral infections, it decreases hepatitis C infection through an anti-lipogenic effect. L-carnitine tartrate has a more than 35-year of proven safety record and is even included in infant formula [24].

In this study, we evaluated the effect of L-carnitine on the expression of key host dependency factors (HDF) in rodent and human tissues and assessed its impact on the SARS-CoV-2 infection. We demonstrate that L-carnitine decreases the levels of ACE2, TMPRSS2, and Furin in rodent tissues and human serum only following exercise-induced inflammation. In lung epithelial cells, L-carnitine reduces the ACE2 levels and significantly limits SARS-CoV-2 infection. These results suggest that L-carnitine may have beneficial effects in mitigating SARS-CoV-2 infection in humans and that L-carnitine supplementation merits further exploration, particularly for vulnerable populations prone to having inflammation.

## 2. Materials and Methods

### 2.1. Materials

L-carnitine tartrate under the form of Carnipure^TM^ tartrate was provided by Lonza Inc., Morristown, NJ, USA.

### 2.2. Cells

Cultured human airway epithelial cells (Calu-3) cells were obtained from the American Type culture Collection (ATCC) and maintained in 50% Dulbecco’s Modified Eagle Medium (DMEM) + 50% Ham’s F12 media supplemented with 10% Fetal Bovine Serum (FBS) and 1% penicillin/streptomycin. Vero E6 cells were maintained in DMEM media supplemented with 10% FBS and 1% penicillin/streptomycin [27].

### 2.3. Animal Tissues

Lung, liver, muscle, and plasma tissues were collected from rats supplemented with different doses of L-carnitine tartrate and subjected to an exercise regimen as previously described [28]. All animal experiments were performed in accordance with the Animal Experimentation Ethics Committee of Firat University (Elazig, Turkey) (2019/140–207).

### 2.4. Human Samples

A total of 80 healthy male and female subjects ranging from 21 to 65 years of age, who were active (i.e., 30 min of moderate activity for 3 days per week) were randomized into two groups (IntegReview, Austin, TX, USA, Protocol #0220). The treatment group was supplemented daily with 2 g of L-carnitine provided from 3 g of Carnipure tartrate (68% L-carnitine and 38% tartaric acid), while the placebo arm received micro-crystalline cellulose a for 5 weeks. Seventy-three subjects completed the trial after seven dropped out due to time constraints. Therefore, data from the 73 were used for descriptive statistics and statistical analysis (NCT04136821). Human sera were obtained from venous blood by venipuncture of the antecubital and collected into a 10 mL EDTA vacutainer tube (BD Vacutainer^®^, Becton, Dickinson and Company, Franklin Lakes, NJ, USA). Blood samples were centrifuged at 770× *g* for 10 min at 4 °C. Then, the resulting serum samples were aliquoted and stored at −80 °C until further analysis. Sera from these subjects were used for the analysis of C-reactive protein (CRP), ACE 1, ACE 2, TMPRSS 2, tumor necrosis factor-alpha (TNF-α), and furin at baseline in the beginning of the trial, at week 5, and 48 h after an exercise challenge as described (Clinicaltrials.gov NCT04420377, 9 June 2020). The latter serves as a surrogate for the inflammation stimulus.

### 2.5. Gene Expression

The expression of rodent genes was measured by qPCR. Total RNA was extracted from frozen tissues samples using an RNeasy 96 Universal kit (Qiagen, Tokyo, Japan) according to the manufacturer’s instructions. cDNA was synthesized from 500 ng of total RNA using the high-capacity reverse transcription cDNA kit containing random primers (Qiagen, Valencia, CA, USA). Real-time quantitative RT-PCR was performed using YBR (Qiagen, Hilden, Germany Catalog No. 330620) and gene-specific primers. Glyceraldehyde-3-phosphate dehydrogenase (GAPDH) was used as an internal control. The primers used for the amplification of rodent genes were ACE1: 5′-AGCATCACCAAGGAGAACTA-3′ (forward), 5′-ACTGGAACTGGATGATGAAG-3′ (reverse), ACE2: 5′-GCTCCTGCTGGCTCCTTCTCA-3′ (forward), 5′-GCCGCAGCCTCGTTCATCTT-3′ (reverse), TMPRSS2: 5′-CACCTGCCATCCACATACAG-3′ (forward), 5′-CCAGAACTTCCAAAGCAAGC-3′ (reverse), Furin: 5′-ACTAA CACTG TGCCC TGGTG GAG-3′ (forward), 5′-ACCCT GGACA GGTAG GTTGG GTA-3′ (reverse), and GAPDH: 5′-GTGGTGAAGCAGGCATCTG-3′ (forward), 5′-GTGGTGAAGCAGGCATCTG-3′ (reverse). To quantify gene expression in human Calu-3, cells were seeded in a 12-well plate (220,000 cells) and treated with 50, 100, 500, 750, or 1000 µM L-carnitine for 24 h. RNA was extracted using TRIzol (Qiagen) as per standard protocol, and total RNA was reverse transcribed using SuperScript II RT (Invitrogen). Expression levels of *ACE1*, *ACE2*, and *TMPRSS2* were evaluated by SYBR real-time PCR using gene-specific primers as previously reported (the ACE1 primers were from [29], while the ACE2 and TMPRSS2 primers were from [11]). Human GAPDH was used as an internal control. The primers for GAPDH were as follows: 5′-GCCATCAATGACCCCTTCATT-3′ (forward) and 5′-TTGACGGTGCCATGGAATTT-3′ (reverse). Fold change in expression relative to that in untreated cells was determined using the standard ddCt method.

### 2.6. Western Blot Using Rodent Tissue Samples

Rodent tissue samples within the same experimental group were pooled for protein analysis. Total proteins (20 μg) were separated by Mini-Protean TGX gel electrophoresis, transferred to a nitrocellulose membrane using the Trans-Blot turbo transfer system (Bio-Rad, Life Sciences Research, Hercules, CA, USA), and probed for ACE1, ACE2, TMPRRS2, and Furin using specific primary antibodies (Santa Cruz Biotechnology, Inc., Dallas, TX, USA). Immunoreactive signals were revealed using horseradish peroxidase-conjugated goat anti-rabbit (Santa Cruz Biotechnology, Inc., Dallas, TX, USA) or goat anti-mouse (Santa Cruz Biotechnology, Inc., Dallas, TX, USA). β-actin was used as an internal control (Santa Cruz Biotechnology, Inc., Dallas, TX, USA). The levels of serum C-reactive protein (CRP) and interleukin 6 (IL-6) were determined using commercially available enzyme-linked immunosorbent assay (ELISA) kits (Abcam, Cambridge, MA, USA) according to the manufacturer instructions and microplate reader (Bio-Tek Elx800 Universal, Bio-Tek Instruments, Inc., Winooski, VT, USA).

### 2.7. ELISA for Human Sera and L-Carnitine Analysis

Human ACE1, ACE2, TMPRSS2, and Furin protein levels were assessed in human sera using commercially available ELISA kits, according to the manufacturer’s recommendations: ACE1 (R&D Systems Inc., Minneapolis, MN, USA), ACE2 (RayBiotech Inc., Peachtree Corners, GA, USA), TMPRSS2 (Novus Biologicals, Littleton, CO, USA), CRP (R&D Systems Inc., Minneapolis, MN, USA), TNF-α (Novus Biologicals), and Furin (Sigma Aldrich, St. Louis, MO, USA). Total, free, and acetyl-carnitine from serum samples were analyzed by HPLC with UV detection according to the manufacturer’s recommendations (Sigma Aldrich, St. Louis, MO, USA).

### 2.8. Effect of L-Carnitine on Cell Viability

Calu-3 cells seeded in a 96-well plate (30,000 cells) were treated with 50, 100, 250, 500, 1000 µM, and 10 mM L-carnitine provided as Carnipure^TM^ tartrate. At 24 h, 48 h, or 72 h post-treatment, media was removed, and cells were subjected to a standard MTT assay according to the manufacturer’s recommendation (Invitrogen; Cat # M6494). Cells treated with vehicle alone were used as a negative control. Absorbance was read at 595 nm using a microplate spectrophotometer. CC_50_ was calculated by non-linear regression using GraphPad Prism V5.0 software (GraphPad Software, Inc., San Diego, CA, USA).

### 2.9. SARS-CoV-2

SARS-CoV-2 virus was originally isolated from a COVID-19 patient in Quebec, Canada and is designated as LSPQ1 variant. The patient virus was amplified and tittered in Vero E6 using plaque assays. All experiments involving infectious SARS-CoV-2 virus were performed in the designated areas of the Biosafety level 3 laboratory previously approved for SARS-CoV-2 work.

### 2.10. Plaque Assays in Vero E6

Vero E6 cells (1.2 × 10^5^ cells/well) were seeded in quadruplicates in 24-well tissue culture plates in Dulbecco’s Modified Eagle Medium (DMEM) supplemented with 10% FBS 36 h before infection. Cells were infected with up to six ten-fold serial dilutions (10^−2^–10^−6^) of viral supernatant containing SARS-CoV-2 for 1 h at 37 °C (200 µL infection volume). The plates were manually rocked every 15 min during the 1 h period. Subsequently, the virus was removed, cells were washed, and overlaying media (containing 0.6% low melt agarose in DMEM with 10% FBS) was added and incubated undisturbed for 60–65 h at 37 °C. Post incubation, cells were fixed with 4% formaldehyde and stained with 0.25% crystal violet (prepared in 30% methanol). High-quality plaque pictures were taken using a high-resolution DLSR camera (Nikon model: D80, objective: AF Micro-Nikkor 60 mm f/2.8D). Plaques were counted manually and in parallel, imaged plaque plates were processed, and plaques were enumerated using an automated algorithm-based Matlab software (developed by the Microscopy platform at the IRCM). Virus titer is expressed as plaque-forming units per ml (PFU/mL, number of plaques × dilution factor of the virus × 1000/volume of virus dilution used for infection (in µL). Multiplicity of infection (MOI) is expressed as: MOI = PFU of virus was used for infection/number of cells.

### 2.11. Effect of L-Carnitine on Cell Infections with SARS-CoV-2

Calu-3 cells were seeded in duplicates in 12-well plates (2.3 × 10^5^ cells/well) and incubated overnight. Cells were pre-treated with various concentrations (0.05–1 mM) of L-carnitine, vehicle alone (DMSO) or 1000 U/mL IFNα-2a for up to 24 h. Thereafter, the cells were infected with SARS-CoV-2 virus at MOI 0.01 and 0.1 for 3 h in 350 µL of serum-free DMEM at 37 °C with the occasional manual rocking of plates. Cells plus media only were used as a control. After incubation, the virus was removed, and the cell monolayer was washed twice successively with PBS and serum-free DMEM. Fresh media (total 1 mL) containing the aforementioned concentrations of L-carnitine was subsequently added to cells. Cell-free supernatant (250 µL) was removed at 12 h, 24 h, and 48 h post-infection. L-carnitine and IFN-2a were replenished at 24 h post-infection. The virus supernatants were stored at −80 °C until further use. Viral production in the supernatant was quantified using a plaque assay on Vero E6 cells as described above. Virus titers (PFU/mL), quantified by the plaque assay done in triplicates, were expressed as mean ± standard deviation.

The percentage of plaques in the presence of L-carnitine was expressed relative to the virus alone group, which was set at 100%. Data were fit using a non-linear regression model, and the equation log inhibitor vs. variable slope (four parameters) was used to determine the half-maximal inhibitory concentration (IC_50_) values in GraphPad Prism V5.0 software (GraphPad Software, Inc., San Diego, CA, USA).

### 2.12. Statistical Analysis

To analyze human serum markers, dependent variables were scrutinized using a two-way mixed analysis of variance (ANOVA) with the condition as the between-group factor (L-carnitine vs. placebo), time as the within-group factor (baseline, week-5 pre- and 48 h post-exercise challenge), and subjects as a random factor. Whenever a significant F-value was obtained, post-hoc testing was performed with a Bonferroni correction for multiple comparisons. For ANOVA procedures, homogeneity of variances and covariances were confirmed by Levene’s test and Box’s M test, respectively. Additionally, Mauchly’s test of sphericity was used to test the assumption of sphericity for two-way interactions. For all analyses, the alpha level was set a priori at *p* < 0.05. Data were presented as mean ± standard error unless otherwise stated. ANOVA and Turkey’s post-hoc test were used to compare the marker changes in the rodent tissue testing. Where appropriate, non-parametric Mann–Whitney’s U-tests (two-tailed) were used to compare ranks between two treatment groups. Human and Calu-3 experiments were analyzed using GraphPad Prism (Prism, San Diego, CA, USA, Version 5 for Calu-3 and Version 8 for human biomarkers) and rodent data by SPSS statistical program (IBM, SPPS Version 21, Armonk, New York: IBM Corp, Armonk, NY, USA).

## 3. Results

### 3.1. Expression Levels of ACE1, ACE2, TMPRSS2, and Furin in Rodent Tissues

**Figure 1:**
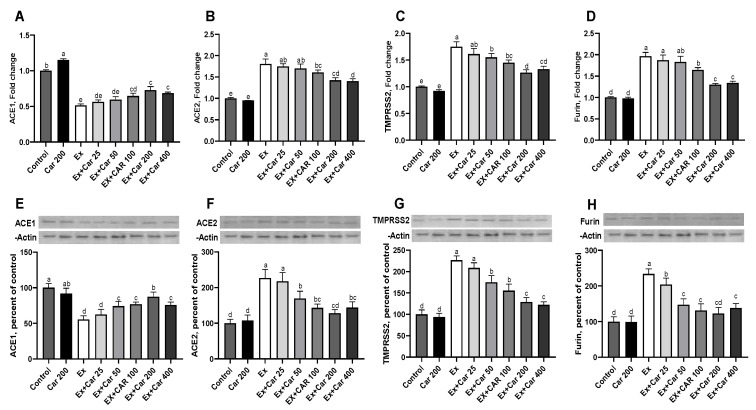
Effect of L-carnitine on the expression of angiotensin-converting enzyme 1 (ACE1), angiotensin-converting enzyme 2 (ACE2), transmembrane protease serine 2 (TMPRSS2), and Furin in rodent lungs. Fold change in the mRNA and protein levels relative to that in the control group: ACE1 (**A**), ACE2 (**B**), TMPRSS2 (**C**), and Furin (**D**) mRNA levels and their respective proteins (**E**–**H**). Protein expression was normalized to that of β-actin. Statistical comparisons are indicated with different superscripts (a–d); in the plots (*p* < 0.05, ANOVA, and Tukey’s post-hoc test). Different letters indicate statistical differences. Shown are mean ± SD of three independent analyses. Control groups: Car: L-carnitine; Ex: Exercise.

**Figure 2:**
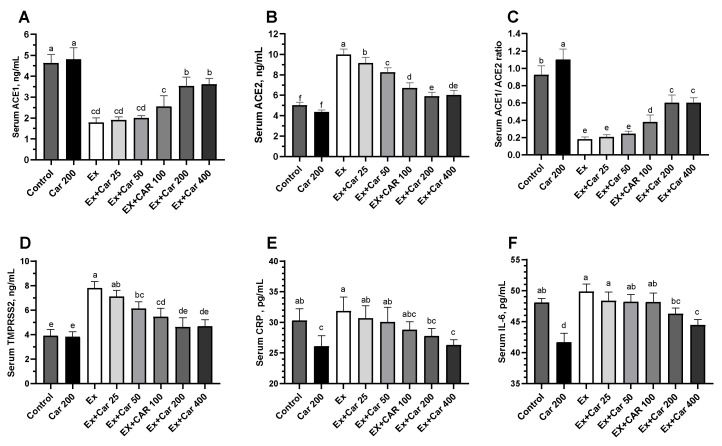
Serum protein levels of ACE1 (**A**), ACE2 (**B**), ACE1/ACE2 ratio (**C**), TMPRSS2 (**D**), C-reactive protein (CRP) (**E**), and interleukin 6 (IL-6) (**F**) in rodents. Statistical comparisons are indicated with different superscripts (a–f); in the plots (*p* < 0.05, ANOVA, and Tukey’s post-hoc test). Different letters indicate statistical differences. Shown are mean ± SD of three independent analyses. Control groups: Car: L-carnitine; Ex: Exercise.

Rodents subjected to a high level training exercise (which serves as an inflammatory stimulus) were supplemented with different doses of L-carnitine by oral gavage for a period of 6 weeks as previously described [28]. The L-carnitine doses used 25, 50, 100, 200 and 400 mg/kg correspond to human doses of 250, 500, 1000, 2000 and 4000 mg per day, respectively. mRNA levels of ACE1, ACE2, TMPRSS2 and Furin were evaluated in the lung, muscle, and liver tissues (Figure 1A–D, Supplementary Figure S1). Compared to control without exercise, the exercise control group showed a statistically significant increase in ACE2, TMPRSS2, and Furin mRNA levels in all tissues (Figure 1A–D, Supplementary Figure S1, about 2 to 3-fold for ACE2 and TMPRSS2 and 1.5 to 2-fold for Furin depending on the tissue). L-carnitine supplementation led to a dose-dependent decrease in ACE2, TMPRSS2 and Furin levels with a maximum effect at the 200 mg/kg dose when compared to the non-exercise levels. ACE1 significantly decreased with exercise and slightly increased with L-carnitine supplementation when combined with exercise and as compared to the exercise control, albeit not reaching the control levels (Figure 1A, Supplementary Figure S1). The protein level assessment mimicked the mRNA effects and showed an increase of ACE2, TMPRSS2, and Furin with exercise and a return to close to the baseline control with L-carnitine supplementation with a maximum effect at 200 mg/kg (Figure 1F–H, Supplementary Figure S2). ACE1 protein levels showed a similar pattern as the mRNA, with a decrease with exercise and an increase with the L-carnitine in the lung, but also in the liver and muscle tissues (Figure 1E, Supplementary Figure S2). In addition, and to compare to the human biomarker data, we also assessed these markers in the sera. Figure 2 shows that L-carnitine supplementation led to 40% to 50% decrease in ACE1, ACE2, and TMPRSS2 proteins when added to an exercise regimen (Figure 2). The serum ACE1/ACE2 ratio was significantly decreased with exercise, and L-carnitine supplementation partly restored this ratio (Figure 2). ACE2, TMPRSS2, and Furin increased and ACE1 decreased upon exercise. These changes coincided with an increased inflammation as determined by CRP and IL-6 inflammatory marker measurements by ELISA during exercise, as shown in Figure 2E,F. L-carnitine supplementation decreased the exercise-induced inflammation as shown by a decrease in CRP and IL-6. The decrease in inflammation by L-carnitine was also observed even without exercise (Figure 2E,F).

### 3.2. Serum ACE1, ACE2, TMPRSS2, Furin, CRP, and TNF-α in Humans

**Figure 3:**
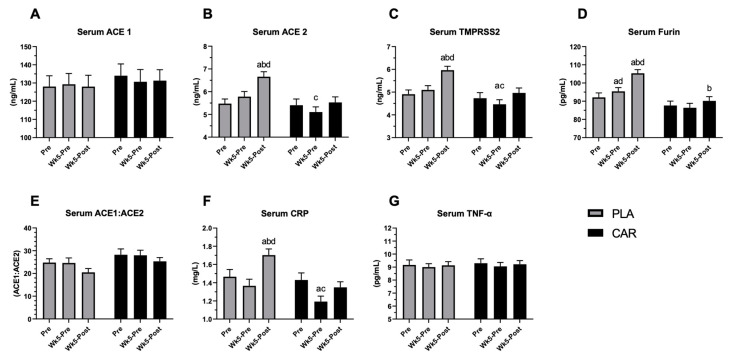
Effect of L-carnitine on serum levels of ACE1, ACE2, TMPRSS2, Furin, CRP, and tumor necrosis factor-alpha (TNF-α) in humans. Serum concentrations of ACE1 (**A**), ACE2 (**B**), TMPRSS2 (**C**), Furin (**D**), ACE1/ACE2 ratio (**E**), CRP (**F**), and TNF-α (**G**) in L-carnitine supplemented (CAR, black bars) or placebo (PLA, gray bars) group at baseline [30], after 5 weeks of supplementation (Wk5-pre) and 48 h after an exercise challenge (Wk5 Post). A significant difference (*p* < 0.05) is indicated, a: different than baseline, b: different than pre-exercise, c: different than post-exercise, d: different between groups.

To assess the effects of L-carnitine on different biomarkers, sera from 73 participants of the human trial were collected at baseline, after 5 weeks of supplementation and 48 h after exercise challenge. Figure 3A shows that neither exercise nor L-carnitine alone affected ACE1 serum levels. On the contrary, the exercise significantly increased the levels of ACE2, Furin, and TMPRSS2 in placebo-treated participants, while in the L-carnitine-treated participants, protein levels remained comparable to the baseline levels before exercise (Figure 3B–D). Post-hoc analysis confirmed that ACE2, TMPRSS2, and Furin rose at 48 h after the exercise challenge compared to baseline and at week 5 in the placebo but not in the L-carnitine treated group. The difference between the groups was significant (Figure 2B–D). Even though there was a significant increase in ACE2 upon exercise without L-carnitine treatment and no change in ACE1 level, the change in the overall ACE1/ACE2 ratio did not reach statistical significance for either group (Figure 3E). The post-hoc analysis revealed that at 5 weeks post-exercise, CRP levels were significantly lower in the L-carnitine-treated group as compared to the placebo-treated group or baseline before exercise (Figure 3F). Neither exercise nor L-carnitine supplementation had an impact on TNF-α levels. All the observed effects were correlated to L-carnitine supplementation. Acetyl-free and total serum carnitine levels increased significantly with supplementation (Supplementary Figure S3).

### 3.3. Effect of L-Carnitine on the Expression of Ace1, Ace2, and Tmprss2 in Human Calu-3 Cells

**Figure 4:**
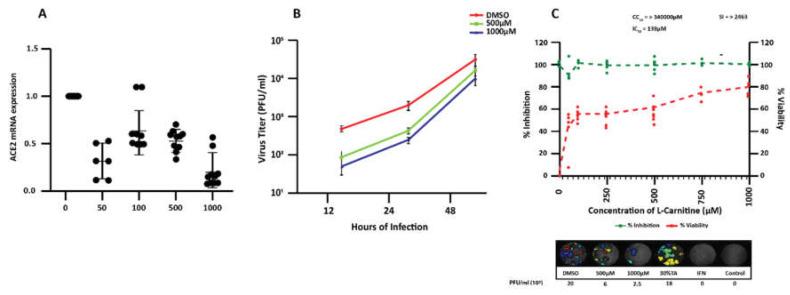
Dose-dependent effect of L-carnitine on ACE2 mRNA and Calu-3 infection by severe acute respiratory syndrome coronavirus 2 (SARS-CoV-2) (**A**) mRNA expression levels of ACE2 in Calu-3 as determined by RT qPCR. Calu-3 cells were left untreated or treated for 24 h with indicated concentrations of L-carnitine. Shown are the mean ± SD of three independent analyses, done in duplicates. Statistical analysis: Mann–Whitney’s test. (**B**) Replication kinetics were studied at 12 h, 24 h, and 48 h post infection by plaque assay to determine infectious virus release from infected cells treated or not with L-carnitine. A line graph represents results of the quadruplicate plaque assay done with two biological replicates (mean ± SD). (**C**) Percentage of inhibition of SARS-CoV-2 titer (at 24 h post infection) in infected cells treated with indicated concentrations of L-carnitine was determined by plaque assay done in quadruplicate (each point represents a replicate) (top panel). The left y-axis indicates the inhibition of virus titer (percent) relative to that of the untreated DMSO-treated group [21]. The right y-axis indicates the cell viability (percent) relative to that of DMSO-treated group (green). The CC_50_ (50% cytotoxic concentration), IC_50_ (half maximal inhibitory concentration), and SI (selectivity index, determined by CC_50_/IC_50_) values for L-carnitine are as shown. Representative plaque images of infected Calu-3 cells treated with indicated varying L-carnitine concentration are shown in the bottom panel. Tartaric acid (TA) 30% and IFN-2a were used as internal controls. *, **, *** statistically significant. ns: not significant.

To assess whether L-carnitine could modulate expression levels of ACE2 and TMPRSS2 in human lung-derived epithelial Calu-3 cells, we first performed a viability assay and found that L-carnitine had no detectable toxicity in this model cell line after the treatment with a range of concentrations (up to 10 mM) for 72 h (Supplementary Figure S4). When Calu-3 cells were exposed to L-carnitine for 24 h, there was a consistent decrease in the level of ACE2 mRNA at all concentrations starting from 50 µM (Figure 4A), with 4.5-fold reduction at 1000 µM (0.22 ± 0.18 relative to 1 in untreated cells). The effect of L-carnitine on ACE1 (Supplementary Figure S5A) and TMPRSS2 (Supplementary Figure S5B) was less conclusive at the different doses tested. In the case of ACE1, only at 100 µM L-carnitine did we observe a statistically significant increase in mRNA expression, while at 1000 µM L-carnitine, the expression was decreased. For TMPRSS2, we observed an increase in mRNA levels at lower L-carnitine concentrations (averaging 1.5 ± 0.3-fold at 50 µM and 5 ± 3.1-fold at 100 µM) but then recorded a significant decrease of more than 2-fold at 500 µM before the expression returned to the level of untreated Calu-3 (Supplementary Figure S5B). L-carnitine did not affect Furin mRNA expression levels (data not shown).

### 3.4. Effect of L-Carnitine on SARS-CoV-2 Infection in Calu-3

Considering L-carnitine decreased ACE2 expression in Calu-3 cells, we assessed whether this reduction in the receptor level altered the susceptibility of Calu-3 cells to SARS-CoV-2 infection. For this purpose, Calu-3 cells were pre-treated for 24 h with varying concentrations of L-carnitine (50–1000 µM) and then infected with the SARS-CoV-2 virus at MOI 0.01. At 12 h and 24 h post-infection, the pre-treatment with 500 µM and 1000 µM L-carnitine resulted in significantly lower viral titer (approximately 1-log) compared to untreated cells (Figure 3B). Our plaque assay analysis at 24 h post-infection revealed that L-carnitine was effective at lowering progeny virus titers by at least 40% at 50 µM and by up to about 80% at 1000 µM (Figure 4C) to give IC_50_ of 138 µM and a selectivity index (SI) of greater than 2463 [31] (Figure 4C).

Overall, the results demonstrate that L-carnitine treatment significantly impairs the susceptibility of Calu-3 cells to SARS-CoV-2 infection, which is most likely through decreasing the expression of viral receptor ACE2 on the cell surface.

## 4. Discussion

The purpose of the present study was to investigate the impact of L-carnitine supplementation on levels of host factors critical for viral entry and pathogenesis (ACE2, TMPRSS2, Furin, ACE1/ACE2 ratio) as well as its effect on SARS-CoV-2 infection in a Calu-3 cell model. First, we found that ACE2, TMPRSS2, and Furin levels in serum and tissues were decreased significantly by L-carnitine but only following exercise-induced inflammation. This decrease coincided with the attenuation of inflammation as shown by decreased CRP levels in rodent and human serum. Then, we assessed the effects of pre-treatment with L-carnitine on SARS-CoV-2 infection in Calu-3, which is a human lung-derived epithelial cell line. We found that L-carnitine pre-treatment decreased Calu-3 susceptibility to infection by SARS-CoV-2, which is most likely through the decrease of viral receptor ACE2 expression and diminished viral attachment. However, in Calu-3, pre-treatment with L-carnitine did not decrease TMPRSS2 and Furin levels. It is possible that these differences observed between the rodent and human biomarkers on the one hand and the Calu-3 cell-based assay may be explained by the lack of inclusion of an inflammatory stimulus in Calu-3 [32]. It is also possible that an additional mechanism of action drove the observed in vivo results beyond inflammation. The L-carnitine’s physiological role is to transport fatty acid through the cellular and mitochondrial membranes leading to their usage as a source of energy by the Krebs cycle in the mitochondria [21]. This enhanced mitochondrial activity was indicated by a decrease in the lactate levels in the rodent serum [28]. It has been recently reported that L-carnitine can physically bind the nuclear receptor HNF4-α and increase its levels [33]. HNF4-α has been shown to decrease ACE2 levels [32,34], providing a potential mechanism for the downregulation of ACE-2 by L-carnitine.

ACE2 levels have been shown to be particularly elevated in alveolar cells, which may account for the viral specificity of lung infections [34]. In addition, inflammatory disease states such as type II diabetes [17], hypertension [2], obesity [35], and general aging and frailty [36,37] are known to be major risk factors for SARS-CoV-2 induced mortality most likely through increased inflammation. It is known that ACE2 levels in lungs and plasma are higher under inflammatory conditions [34], thus increasing the susceptibility of cells from the population suffering from the above conditions to SARS-CoV-2 infection. L-carnitine has been reported to play a regulatory role in inflammatory processes [22,23,38], and L-carnitine supplementation has beneficial effects in populations with relatively low L-carnitine levels, such as that which occurs in the elderly and in a variety of inflammatory disease states [25].

Both exhaustive aerobic and resistance exercises have been shown to transiently (<96 h) increase the oxidative stress and mechanical damage in muscles [39], thereby enhancing inflammation [40]. Exercise-induced inflammation can augment ACE2 levels [41], leading to reduced inflammation through generating the anti-inflammatory Angiotensin-(1–7) [14]. Indeed, CRP concentration elevates to 168% above the baseline as a result of exercise [42], peaking at 253% 2 days after [42]. In our studies, the exercise-induced inflammation also resulted in an increase in ACE2 and CRP serum levels, which was mitigated by L-carnitine supplementation. Interestingly, athletes have been reported to be at high risk of complications from SARS-CoV-2 infection, leading ultimately to lung and cardiac injuries [43]. It is plausible that this increased susceptibility of athletes to SARS-CoV-2 complications is linked to the exercise-linked increased levels of the ACE2 receptor in lung and muscle cells. Future studies may explore if a potential additional benefit of L-carnitine supplementation exists that lowers the susceptibility of this population to SARS-CoV-2 infection.

The physiological role of ACE2 is to lower blood pressure and counteract inflammation by converting pro-inflammatory Angiotensin II to anti-inflammatory Angiotensin (1–7) [5]. The conversion from Angiotensin I to Angiotensin II is mediated by ACE1. Increases in ACE1, or a decrease in ACE2, may reflect an increased inflammatory state characteristic of advanced diseased COVID-19 patients who experience the deleterious cytokine storm [36]. Indeed, it has been reported that patients with hypertension and treated with ACE1 or angiotensin receptor blocker (ARB) medications, targeting the Renin-Angiotensin System (RAS), may have the ACE1/ACE2 ratio elevated, leading potentially to a worsening of SARS-CoV-2 infection [44]. Therefore, therapies attempting to depress ACE2 should be done in a way that either lowers or does not elevate the ACE1/ACE2 ratio [36]. We found that L-carnitine shifted the ACE1/ACE2 ratio in lungs and muscle tissue to a more anti-inflammatory state while keeping the same ratio in humans. These data raise the possibility that L-carnitine may potentially alleviate inflammation-induced muscle weakness and lung injuries seen in humans. Finally, our data show that L-carnitine pre-treatment decreases ACE2 expression and inhibits the production of infectious viral particles in Calu-3 lung epithelial cells.

## 5. Conclusions

The present study provides evidence that L-carnitine supplementation reduces the expression of SARS-CoV-2 receptor ACE2 and proteases required for viral entry (TMPRSS2 and Furin) following exercise-induced inflammation in rodents and humans. L-carnitine supplementation also significantly decreases the susceptibility of human lung–epithelial cells to the infection by SARS-CoV-2. Considering L-carnitine has an excellent safety record over 35 years of use [24], future investigations of potentially beneficial effects of L-carnitine in preventing SARS-CoV-2 infection and complications in humans are warranted. However, some of the limitations of the current study include the lack of effects on TMPRSS2 and Furin in Calu-3 cells despite the observed decrease in human and rodent tissues. This points to the limitation of translating the cell-based assays to the rodent and human findings. While L-carnitine treatment reduced the susceptibility of human lung epithelial cells to SARS-CoV-2 infection in vitro, whether this effect is solely the result of ACE2 downregulation or also an impairment of viral entry remains to be determined.

## Data Availability

Data are contained within this article and supplementary material.

## Supplementary Materials

The following are available online at https://www.mdpi.com/article/10.3390/nu13041297/s1, Figure S1: Effect of L-carnitine on mRNA of ACE1, ACE2, TMPRSS2 and Furin in rodent muscle and liver tissues. Figure S2: Effect of L-carnitine on of ACE1, ACE2, TMPRSS2, and Furin in rodent muscle and liver tissues. Figure S3: Effect of L-carnitine supplementation on acetyl-, free and total carnitine levels in human serum. Figure S4: Effect of L-carnitine on Calu-3 cell viability. Figure S5: Dose-dependent effect of L-carnitine on the expression of ACE1 and TMPRSS2 in Calu-3 cells.
