# Supplementary material for: L-Carnitine Tartrate Downregulates the ACE2 Receptor and Limits SARS-CoV-2 Infection
Source: Nutrients. 2021 Apr 14;13(4):1297. doi: 10.3390/nu13041297 (PMC8071056; doi:10.3390/nu13041297)
Supplement: Supplementary file 1 [file nutrients-13-01297-s001.pdf]

Supplement Figures 1-5

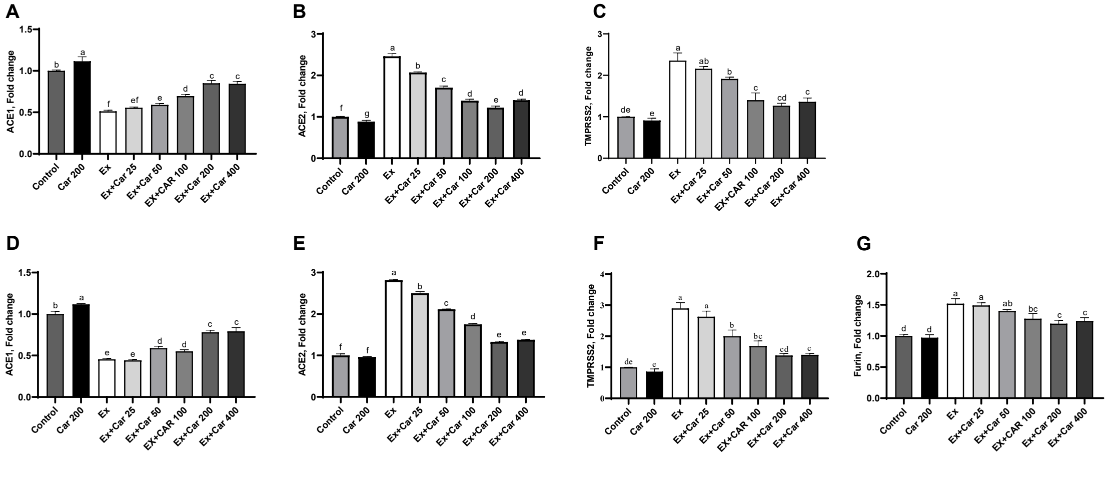

Supplemental 1: Effect of L-carnitine on ACE1, ACE2, TMPRSS2 and Furin expression in rodent muscle and liver tissues

Fold change in the mRNA levels from control for ACE1 (A), ACE2 (B) and TMPRSS2 (C) in muscle and ACE1 (D), ACE2 (E), TMPRSS2 (F) and Furin (G) in liver. Statistical comparisons are indicated with different superscripts (a-g); in the plots ($p < 0.05$, ANOVA, and Tukey's post-hoc test). Shown are mean ± SD of 3 independent analyses. Different letters indicate statistical differences. Shown are mean ± SD of 3 independent analyses. Car: L-carnitine; Ex: Exercise

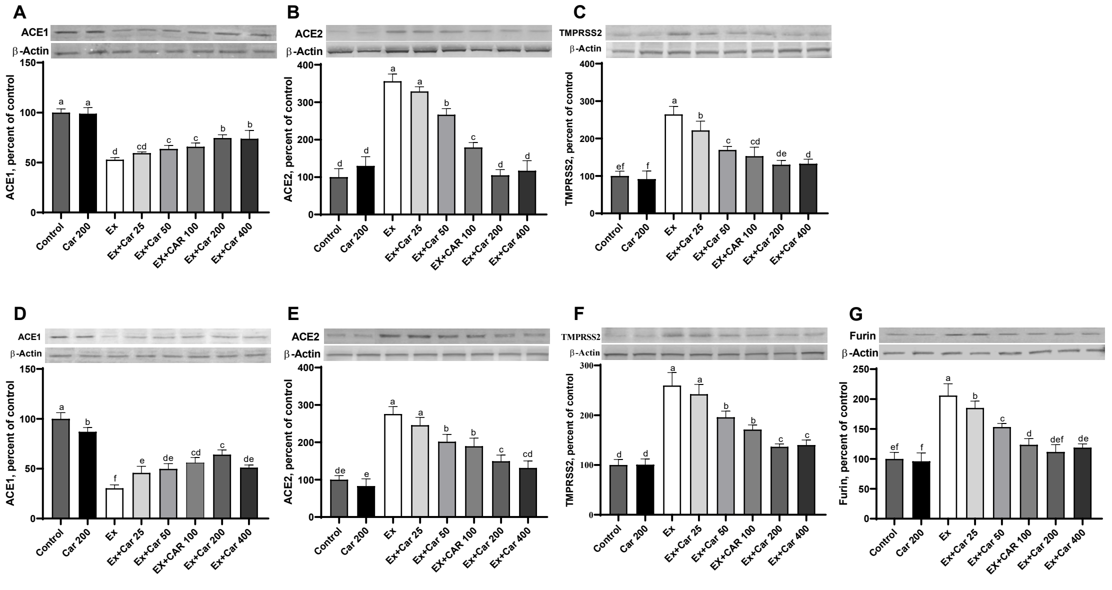

Supplemental 2: Effect of L-carnitine on ACE1, ACE2, TMPRSS2 and Furin expression in rodent muscle and liver tissues

Protein quantification based on densitometric analysis and adjusted with b-actin for ACE1 (A), ACE2 (B) and TMPRSS2 (C) in muscle and ACE1 (D), ACE2 (E), TMPRSS2 (F) and Furin (G) in liver. Statistical comparisons are indicated with different superscripts (a-g); in the plots ($p < 0.05$,

ANOVA, and Tukey's post-hoc test). Different letters indicate statistical differences. Shown are mean ± SD of 3 independent analyses. Car: L-carnitine; Ex: Exercise

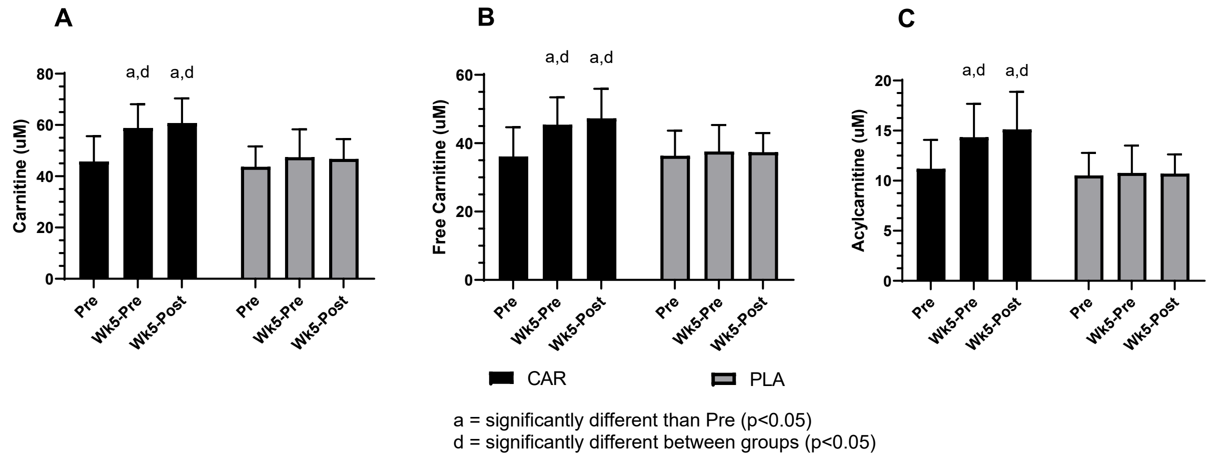

Supplemental 3: Serum Concentrations for Total Carnitine (A), Free Carnitine (B), and Acylcarnitine (C).

Serum levels were measured in humans who were supplemented with L-carnitine (CAR, black bars) or placebo (PLA, grey bars) group at pre supplementation (Pre), after 5 weeks of supplementation (Wk5-pre) and 48 h after an exercise challenge (Wk5 Post). Significant difference ($p < 0.05$) is indicated, a: different than pre supplementation, d: different between groups.

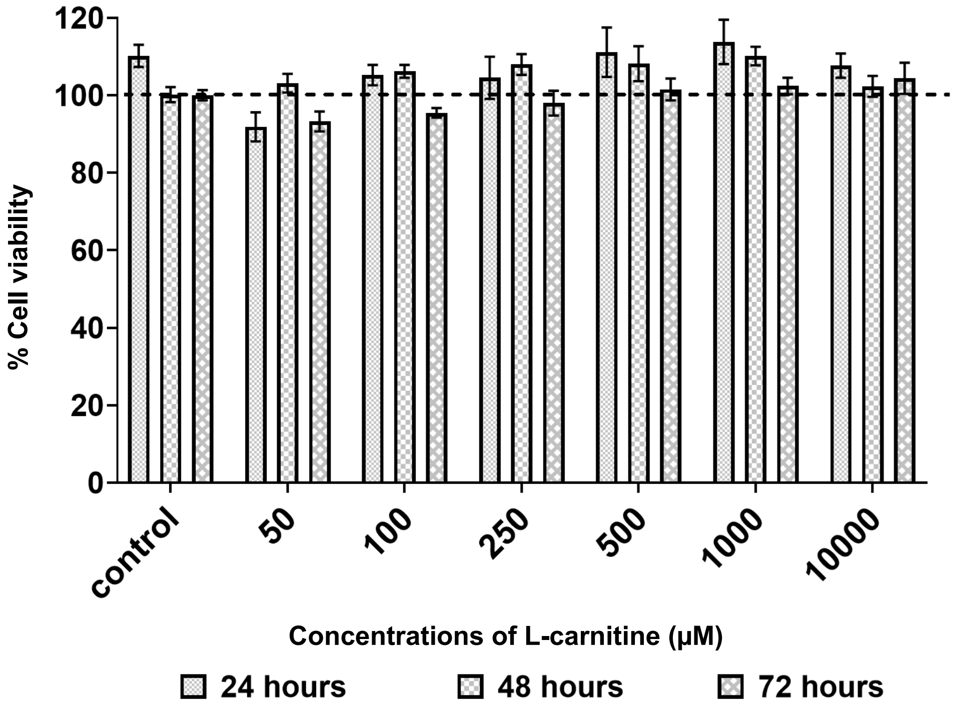

Supplemental 4: Effect of L-carnitine on Calu-3 cell viability

Cells were treated with 50, 100, 250, 500, 1,000 and 10,000 μM of L-carnitine for 24, 48 and 72h. Viability was tested using MTT assay

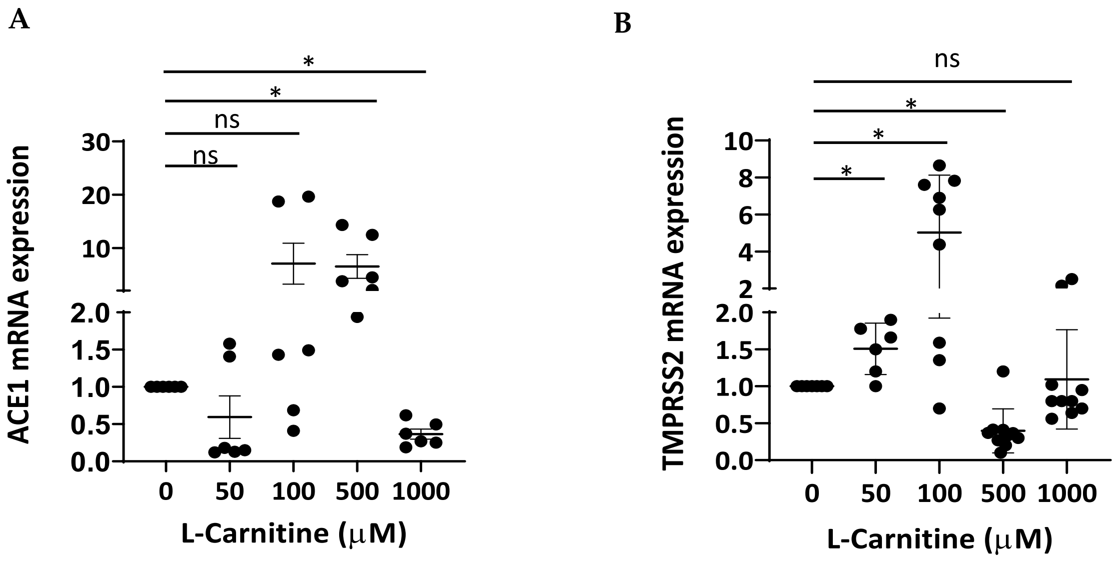

Supplemental 5: Dose-dependent effect of L-carnitine on ACE1 and TMPRSS2 mRNA levels in Calu-3 cells

mRNA expression levels of ACE1 (A) and TMPRSS2 (B) in Calu-3 was determined by RT qPCR. Calu-3 cells were left untreated or treated for 24 h with indicated concentrations of L-carnitine. Shown are mean ± SD of 3 independent analyses, done in duplicates. Statistical analysis: Mann-Whitney's test.** significant, ns not significant compared to untreated
